# 9-Benzyl-3-bromo-9*H*-carbazole

**DOI:** 10.1107/S1600536809024969

**Published:** 2009-07-04

**Authors:** Peng-Mian Huang, Xiao-Chun Wang

**Affiliations:** a College of Chemistry & Bioengineering, Changsha University of Science & Technology, Changsha 410076, People’s Republic of China; b Department of Clinical Laboratory, XiangYa Medical College of Central South University, Changsha 410013, People’s Republic of China

## Abstract

The title compound, C_19_H_14_BrN, was synthesized by the *N*-alkyl­ation of (chloro­meth­yl)benzene with 3-bromo-9*H*-carbazole. The carbazole ring system is essentially planar (r.m.s. deviation = 0.013 Å) and forms a dihedral angle of 87.1 (2)° with the phenyl ring.

## Related literature

For the synthesis, see: Duan *et al.* (2005 ▶). For the pharmaceutical properties of *N*-alkyl carbazoles, see: Buu-Hoï & Royer (1950 ▶).
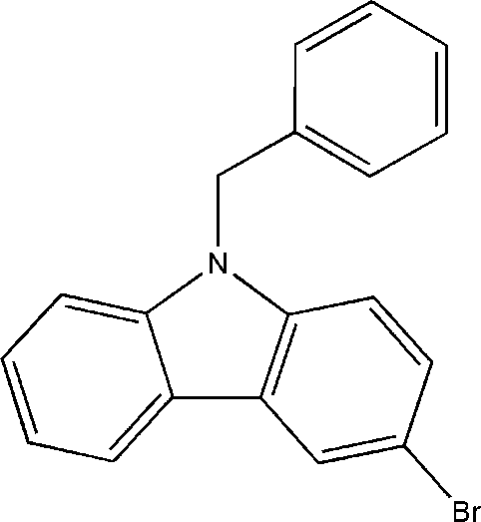


## Experimental

### 

#### Crystal data

C_19_H_14_BrN
*M* *_r_* = 336.22
Orthorhombic, 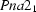

*a* = 17.629 (3) Å
*b* = 14.666 (2) Å
*c* = 5.6420 (8) Å
*V* = 1458.7 (4) Å^3^
*Z* = 4
Mo *K*α radiation
μ = 2.81 mm^−1^
*T* = 113 K
0.14 × 0.12 × 0.10 mm

#### Data collection

Rigaku Saturn diffractometer
Absorption correction: multi-scan (*CrystalClear*; Rigaku/MSC, 2005 ▶) *T*_min_ = 0.682, *T*_max_ = 0.755
14426 measured reflections
3441 independent reflections
2801 reflections with *I* > 2σ(*I*)
*R*_int_ = 0.045

#### Refinement

*R*[*F* ^2^ > 2σ(*F* ^2^)] = 0.036
*wR*(*F* ^2^) = 0.069
*S* = 0.98
3441 reflections
191 parameters
1 restraint
H-atom parameters constrained
Δρ_max_ = 0.95 e Å^−3^
Δρ_min_ = −0.52 e Å^−3^
Absolute structure: Flack (1983 ▶), 1521 Friedel pairs
Flack parameter: −0.005 (10)

### 

Data collection: *CrystalClear* (Rigaku/MSC, 2005 ▶); cell refinement: *CrystalClear*; data reduction: *CrystalClear*; program(s) used to solve structure: *SHELXS97* (Sheldrick, 2008 ▶); program(s) used to refine structure: *SHELXL97* (Sheldrick, 2008 ▶); molecular graphics: *SHELXTL* (Sheldrick, 2008 ▶); software used to prepare material for publication: *SHELXTL*.

## Supplementary Material

Crystal structure: contains datablocks I, global. DOI: 10.1107/S1600536809024969/hb5016sup1.cif

Structure factors: contains datablocks I. DOI: 10.1107/S1600536809024969/hb5016Isup2.hkl

Additional supplementary materials:  crystallographic information; 3D view; checkCIF report

## Figures and Tables

**Fig. 1.**
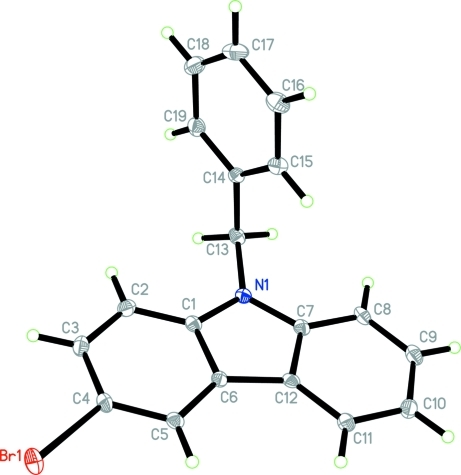
Molecular structure of (I) with displacement ellipsoids drawn at the 30% probability level. H atoms are presented as spheres of arbitrary radius.

## supplementary crystallographic information

### Comment

N-Alkyl carbazoles possess valuable pharmaceutical properties (Buu-Hoï & Royer, 1950). In this paper, the synthesis and the crystal structure of the title compound, (I), is reported.

The carbazole ring is essentially planar, with a r.m.s. deviation from the mean plane of 0.013 Å for the non-hydrogen atoms. The dihedral angle formed between the carbazole unit and the benzene ring is 92.9 (2) Å.

### Experimental

The title compound was prepared according to the procedure of Duan *et al.* (2005).  The title compound (40 mg) was dissolved in a mixture of chloroform (5 ml) and ethanol (7 ml) and the solution was kept at room temperature for 11 days. Evaporation of the solution gave colourless blocks of (I).

### Refinement

All H atoms were included in idealized positions (C—H = 0.95–0.99Å) and refined as riding with *U*_iso_(H) = 1.2*U*_eq_(C).

### Crystal data

**Table:**

C_19_H_14_BrN	*D*_x_ = 1.531 Mg m^−^^3^
*M**_r_* = 336.22	Melting point = 392–394 K
Orthorhombic, *P**n**a*2_1_	Mo *K*α radiation, λ = 0.71070 Å
Hall symbol: P 2c -2n	Cell parameters from 3798 reflections
*a* = 17.629 (3) Å	θ = 1.8–27.9°
*b* = 14.666 (2) Å	µ = 2.81 mm^−^^1^
*c* = 5.6420 (8) Å	*T* = 113 K
*V* = 1458.7 (4) Å^3^	Block, colorless
*Z* = 4	0.14 × 0.12 × 0.10 mm
*F*(000) = 680	

### Data collection

**Table:**

Rigaku Saturn diffractometer	3441 independent reflections
Radiation source: rotating anode	2801 reflections with *I* > 2σ(*I*)
confocal multilayer X-ray optic	*R*_int_ = 0.045
Detector resolution: 7.31 pixels mm^-1^	θ_max_ = 27.9°, θ_min_ = 1.8°
ω and φ scans	*h* = −23→23
Absorption correction: multi-scan (*CrystalClear*; Rigaku/MSC, 2005)	*k* = −19→19
*T*_min_ = 0.682, *T*_max_ = 0.755	*l* = −7→7
14426 measured reflections	

### Refinement

**Table:**

Refinement on *F*^2^	Hydrogen site location: inferred from neighbouring sites
Least-squares matrix: full	H-atom parameters constrained
*R*[*F*^2^ > 2σ(*F*^2^)] = 0.036	*w* = 1/[σ^2^(*F*_o_^2^) + (0.0241*P*)^2^] where *P* = (*F*_o_^2^ + 2*F*_c_^2^)/3
*wR*(*F*^2^) = 0.069	(Δ/σ)_max_ = 0.001
*S* = 0.98	Δρ_max_ = 0.95 e Å^−^^3^
3441 reflections	Δρ_min_ = −0.52 e Å^−^^3^
191 parameters	Extinction correction: *SHELXL97* (Sheldrick, 2008), Fc^*^=kFc[1+0.001xFc^2^λ^3^/sin(2θ)]^-1/4^
1 restraint	Extinction coefficient: 0.0148 (7)
Primary atom site location: structure-invariant direct methods	Absolute structure: Flack (1983), 1521 Friedel pairs
Secondary atom site location: difference Fourier map	Flack parameter: −0.005 (10)

### Special details

**Table:**

Geometry. All esds (except the esd in the dihedral angle between two l.s. planes) are estimated using the full covariance matrix. The cell esds are taken into account individually in the estimation of esds in distances, angles and torsion angles; correlations between esds in cell parameters are only used when they are defined by crystal symmetry. An approximate (isotropic) treatment of cell esds is used for estimating esds involving l.s. planes.
Refinement. Refinement of F^2^ against ALL reflections. The weighted R-factor wR and goodness of fit S are based on F^2^, conventional R-factors R are based on F, with F set to zero for negative F^2^. The threshold expression of F^2^ > 2sigma(F^2^) is used only for calculating R-factors(gt) etc. and is not relevant to the choice of reflections for refinement. R-factors based on F^2^ are statistically about twice as large as those based on F, and R- factors based on ALL data will be even larger.

### Fractional atomic coordinates and isotropic or equivalent isotropic displacement parameters (Å^2^)

**Table:**

	*x*	*y*	*z*	*U*_iso_*/*U*_eq_	
Br1	0.257493 (13)	0.192950 (18)	0.66598 (14)	0.02943 (11)	
N1	0.53686 (13)	0.19508 (14)	0.0539 (4)	0.0196 (5)	
C1	0.46874 (12)	0.20289 (15)	0.1699 (6)	0.0180 (5)	
C2	0.40547 (16)	0.25415 (17)	0.1164 (5)	0.0229 (7)	
H2	0.4049	0.2918	−0.0206	0.027*	
C3	0.34289 (16)	0.25037 (19)	0.2638 (5)	0.0231 (7)	
H3	0.2988	0.2849	0.2278	0.028*	
C4	0.34503 (16)	0.19535 (18)	0.4663 (5)	0.0206 (7)	
C5	0.40726 (15)	0.14351 (19)	0.5264 (5)	0.0191 (6)	
H5	0.4072	0.1064	0.6643	0.023*	
C6	0.47028 (15)	0.14744 (17)	0.3781 (5)	0.0176 (6)	
C7	0.58394 (13)	0.13641 (16)	0.1792 (6)	0.0191 (6)	
C8	0.65823 (14)	0.10891 (17)	0.1344 (6)	0.0228 (7)	
H8	0.6848	0.1303	−0.0013	0.027*	
C9	0.69198 (16)	0.0502 (2)	0.2913 (5)	0.0269 (7)	
H9	0.7424	0.0303	0.2617	0.032*	
C10	0.65424 (18)	0.0186 (2)	0.4947 (5)	0.0260 (7)	
H10	0.6794	−0.0215	0.6010	0.031*	
C11	0.58048 (16)	0.04587 (18)	0.5403 (5)	0.0222 (7)	
H11	0.5547	0.0246	0.6774	0.027*	
C12	0.54435 (15)	0.10506 (18)	0.3825 (5)	0.0179 (6)	
C13	0.56053 (16)	0.25418 (18)	−0.1405 (5)	0.0224 (7)	
H13A	0.5185	0.2588	−0.2563	0.027*	
H13B	0.6042	0.2258	−0.2223	0.027*	
C14	0.58274 (15)	0.34943 (19)	−0.0620 (5)	0.0197 (6)	
C15	0.62312 (14)	0.36493 (17)	0.1461 (6)	0.0241 (6)	
H15	0.6360	0.3154	0.2470	0.029*	
C16	0.64439 (15)	0.4530 (2)	0.2054 (6)	0.0307 (8)	
H16	0.6726	0.4631	0.3464	0.037*	
C17	0.6255 (2)	0.5257 (2)	0.0643 (6)	0.0344 (9)	
H17	0.6402	0.5858	0.1077	0.041*	
C18	0.58455 (17)	0.5108 (2)	−0.1427 (7)	0.0336 (8)	
H18	0.5713	0.5605	−0.2424	0.040*	
C19	0.56306 (16)	0.42221 (19)	−0.2030 (6)	0.0261 (7)	
H19	0.5345	0.4120	−0.3432	0.031*	

### Atomic displacement parameters (Å^2^)

**Table:**

	*U*^11^	*U*^22^	*U*^33^	*U*^12^	*U*^13^	*U*^23^
Br1	0.01928 (15)	0.03967 (17)	0.02934 (18)	0.00396 (12)	0.0029 (2)	−0.0045 (2)
N1	0.0176 (13)	0.0205 (12)	0.0208 (13)	−0.0014 (10)	0.0012 (11)	0.0036 (11)
C1	0.0175 (12)	0.0180 (12)	0.0184 (14)	−0.0031 (10)	−0.002 (2)	−0.0038 (16)
C2	0.0249 (15)	0.0199 (14)	0.024 (2)	−0.0015 (12)	−0.0055 (13)	0.0013 (13)
C3	0.0202 (16)	0.0211 (15)	0.0279 (17)	0.0034 (13)	−0.0056 (13)	−0.0036 (13)
C4	0.0152 (15)	0.0237 (16)	0.0228 (16)	0.0004 (12)	0.0026 (13)	−0.0038 (13)
C5	0.0179 (15)	0.0189 (14)	0.0204 (16)	−0.0040 (12)	−0.0021 (12)	−0.0011 (12)
C6	0.0166 (15)	0.0150 (14)	0.0213 (15)	−0.0022 (11)	−0.0031 (13)	−0.0029 (12)
C7	0.0185 (12)	0.0173 (12)	0.0214 (14)	−0.0035 (10)	−0.0027 (18)	−0.0048 (16)
C8	0.0159 (13)	0.0259 (14)	0.0268 (19)	−0.0044 (11)	0.0002 (14)	−0.0051 (15)
C9	0.0186 (16)	0.0258 (17)	0.0364 (19)	−0.0017 (13)	−0.0015 (15)	−0.0078 (15)
C10	0.0228 (17)	0.0225 (15)	0.0326 (18)	0.0020 (13)	−0.0053 (14)	0.0002 (14)
C11	0.0201 (16)	0.0231 (15)	0.0233 (16)	−0.0031 (12)	−0.0031 (13)	−0.0015 (13)
C12	0.0178 (15)	0.0140 (13)	0.0217 (16)	−0.0042 (11)	0.0010 (13)	−0.0034 (12)
C13	0.0195 (16)	0.0267 (16)	0.0209 (15)	−0.0024 (12)	0.0024 (13)	0.0004 (14)
C14	0.0170 (15)	0.0208 (14)	0.0215 (17)	0.0003 (12)	0.0076 (13)	0.0035 (14)
C15	0.0272 (14)	0.0229 (14)	0.0222 (16)	−0.0034 (11)	0.0000 (18)	0.0000 (17)
C16	0.0311 (16)	0.0336 (17)	0.028 (2)	−0.0080 (13)	0.0013 (15)	−0.0054 (16)
C17	0.039 (2)	0.0210 (16)	0.043 (2)	−0.0068 (15)	0.0162 (16)	−0.0033 (16)
C18	0.0319 (19)	0.0247 (16)	0.044 (2)	0.0040 (14)	0.0113 (18)	0.0080 (16)
C19	0.0213 (16)	0.0324 (17)	0.0244 (17)	0.0004 (13)	0.0045 (14)	0.0051 (14)

### Geometric parameters (Å, °)

**Table:**

Br1—C4	1.911 (3)	C9—H9	0.9500
N1—C1	1.373 (3)	C10—C11	1.385 (4)
N1—C7	1.389 (3)	C10—H10	0.9500
N1—C13	1.459 (3)	C11—C12	1.397 (4)
C1—C2	1.379 (3)	C11—H11	0.9500
C1—C6	1.429 (4)	C13—C14	1.517 (4)
C2—C3	1.382 (4)	C13—H13A	0.9900
C2—H2	0.9500	C13—H13B	0.9900
C3—C4	1.399 (4)	C14—C19	1.376 (4)
C3—H3	0.9500	C14—C15	1.392 (4)
C4—C5	1.377 (4)	C15—C16	1.385 (4)
C5—C6	1.392 (4)	C15—H15	0.9500
C5—H5	0.9500	C16—C17	1.373 (4)
C6—C12	1.446 (3)	C16—H16	0.9500
C7—C8	1.393 (3)	C17—C18	1.390 (5)
C7—C12	1.419 (4)	C17—H17	0.9500
C8—C9	1.371 (4)	C18—C19	1.395 (4)
C8—H8	0.9500	C18—H18	0.9500
C9—C10	1.405 (4)	C19—H19	0.9500
			
C1—N1—C7	109.4 (2)	C9—C10—H10	120.0
C1—N1—C13	124.0 (2)	C10—C11—C12	119.3 (3)
C7—N1—C13	125.4 (2)	C10—C11—H11	120.4
N1—C1—C2	130.5 (3)	C12—C11—H11	120.4
N1—C1—C6	109.1 (2)	C11—C12—C7	119.5 (2)
C2—C1—C6	120.4 (3)	C11—C12—C6	133.6 (3)
C1—C2—C3	119.5 (3)	C7—C12—C6	106.9 (2)
C1—C2—H2	120.3	N1—C13—C14	113.7 (2)
C3—C2—H2	120.3	N1—C13—H13A	108.8
C2—C3—C4	119.5 (3)	C14—C13—H13A	108.8
C2—C3—H3	120.2	N1—C13—H13B	108.8
C4—C3—H3	120.2	C14—C13—H13B	108.8
C5—C4—C3	122.8 (3)	H13A—C13—H13B	107.7
C5—C4—Br1	119.2 (2)	C19—C14—C15	119.3 (3)
C3—C4—Br1	118.1 (2)	C19—C14—C13	118.7 (3)
C4—C5—C6	117.7 (3)	C15—C14—C13	121.9 (2)
C4—C5—H5	121.2	C16—C15—C14	119.6 (3)
C6—C5—H5	121.2	C16—C15—H15	120.2
C5—C6—C1	120.2 (2)	C14—C15—H15	120.2
C5—C6—C12	133.8 (3)	C17—C16—C15	121.3 (3)
C1—C6—C12	106.0 (2)	C17—C16—H16	119.4
N1—C7—C8	130.4 (3)	C15—C16—H16	119.4
N1—C7—C12	108.6 (2)	C16—C17—C18	119.4 (3)
C8—C7—C12	121.0 (3)	C16—C17—H17	120.3
C9—C8—C7	118.2 (3)	C18—C17—H17	120.3
C9—C8—H8	120.9	C17—C18—C19	119.5 (3)
C7—C8—H8	120.9	C17—C18—H18	120.2
C8—C9—C10	121.9 (3)	C19—C18—H18	120.2
C8—C9—H9	119.0	C14—C19—C18	120.9 (3)
C10—C9—H9	119.0	C14—C19—H19	119.6
C11—C10—C9	120.1 (3)	C18—C19—H19	119.6
C11—C10—H10	120.0		
			
C7—N1—C1—C2	178.6 (3)	C8—C9—C10—C11	0.7 (4)
C13—N1—C1—C2	10.5 (4)	C9—C10—C11—C12	−0.1 (4)
C7—N1—C1—C6	−0.3 (3)	C10—C11—C12—C7	−0.3 (4)
C13—N1—C1—C6	−168.5 (2)	C10—C11—C12—C6	−178.6 (3)
N1—C1—C2—C3	−179.9 (3)	N1—C7—C12—C11	−179.0 (2)
C6—C1—C2—C3	−1.1 (4)	C8—C7—C12—C11	0.1 (4)
C1—C2—C3—C4	0.7 (4)	N1—C7—C12—C6	−0.2 (3)
C2—C3—C4—C5	−0.4 (4)	C8—C7—C12—C6	178.9 (2)
C2—C3—C4—Br1	179.5 (2)	C5—C6—C12—C11	−1.5 (5)
C3—C4—C5—C6	0.5 (4)	C1—C6—C12—C11	178.5 (3)
Br1—C4—C5—C6	−179.48 (19)	C5—C6—C12—C7	180.0 (3)
C4—C5—C6—C1	−0.8 (4)	C1—C6—C12—C7	0.0 (3)
C4—C5—C6—C12	179.2 (3)	C1—N1—C13—C14	73.6 (3)
N1—C1—C6—C5	−179.8 (2)	C7—N1—C13—C14	−92.7 (3)
C2—C1—C6—C5	1.2 (4)	N1—C13—C14—C19	−141.7 (3)
N1—C1—C6—C12	0.2 (3)	N1—C13—C14—C15	39.1 (4)
C2—C1—C6—C12	−178.9 (2)	C19—C14—C15—C16	−1.5 (4)
C1—N1—C7—C8	−178.7 (3)	C13—C14—C15—C16	177.7 (3)
C13—N1—C7—C8	−10.7 (4)	C14—C15—C16—C17	0.9 (4)
C1—N1—C7—C12	0.3 (3)	C15—C16—C17—C18	−0.4 (5)
C13—N1—C7—C12	168.3 (2)	C16—C17—C18—C19	0.3 (5)
N1—C7—C8—C9	179.3 (3)	C15—C14—C19—C18	1.5 (4)
C12—C7—C8—C9	0.4 (4)	C13—C14—C19—C18	−177.7 (3)
C7—C8—C9—C10	−0.8 (4)	C17—C18—C19—C14	−0.9 (4)
